# Ten Years of Surveillance for Invasive *Streptococcus pneumoniae* during the Era of Antiretroviral Scale-Up and Cotrimoxazole Prophylaxis in Malawi

**DOI:** 10.1371/journal.pone.0017765

**Published:** 2011-03-15

**Authors:** Dean B. Everett, Mavuto Mukaka, Brigitte Denis, Stephen B. Gordon, Enitan D. Carrol, Joep J. van Oosterhout, Elizabeth M. Molyneux, Malcolm Molyneux, Neil French, Robert S. Heyderman

**Affiliations:** 1 Malawi-Liverpool-Wellcome Trust Clinical Research Programme, College of Medicine, Chichiri, Blantyre, Malawi; 2 University of Liverpool, Liverpool, United Kingdom; 3 The Liverpool School of Tropical Medicine, Liverpool, United Kingdom; 4 Institute of Child Health, University of Liverpool, Liverpool, United Kingdom; 5 Department of Medicine, College of Medicine, Blantyre, Malawi; 6 Department of Paediatrics, College of Medicine, Blantyre, Malawi; 7 The London School of Hygiene and Tropical Medicine, London, United Kingdom; Massachusetts General Hospital, United States of America

## Abstract

**Objective:**

To document trends in invasive pneumococcal disease (IPD) in a central hospital in Malawi during the period of national scale-up of antiretroviral therapy (ART) and cotrimoxazole prophylaxis.

**Methods:**

Between 1 January 2000 and 31 December 2009 almost 100,000 blood cultures and 40,000 cerebrospinal fluid (CSF) cultures were obtained from adults and children admitted to the Queen Elizabeth Central Hospital, Blantyre, Malawi with suspected severe bacterial infection.

**Results:**

4,445 pneumococcal isolates were obtained over the 10 year period. 1,837 were from children: 885 (19.9%) from blood and 952 (21.4%) from CSF. 2,608 were from adults: 1,813 (40.8%) from blood and 795 (17.9%) from CSF. At the start of the surveillance period cotrimoxazole resistance was 73.8% and at the end was 92.6%. Multidrug resistance (MDR) was present in almost one third of isolates and was constant over time. Free ART was introduced in Malawi in 2004. From 2005 onwards there was a decline in invasive pneumococcal infections with a negative correlation between ART scale-up and the decline in IPD (Pearson's correlation r = −0.91; p<0.001).

**Conclusion:**

During 2004–2009, national ART scale-up in Malawi was associated with a downward trend in IPD at QECH. The introduction of cotrimoxazole prophylaxis in HIV-infected groups has not coincided with a further increase in pneumococcal cotrimoxazole or multidrug resistance. These data highlight the importance of surveillance for high disease burden infections such as IPD in the region, which will be vital for monitoring pneumococcal conjugate vaccine introduction into national immunisation programmes.

## Introduction

Approximately 1.1 million deaths annually are attributed to invasive pneumococcal disease (IPD) worldwide, accounting for 9% of all deaths in developing countries [1], [2], [3], [4]. Pneumococcal meningitis and bloodstream infections are frequently HIV-related, especially in adults, and are associated with a high mortality [5], [6], [7], [8], [9].

In Malawi, *S. pneumoniae* is a leading cause of pneumonia, sepsis, bacterial meningitis and death in both children and adults [6], [8], [10]. Malawi is a landlocked country in southern Africa that lies to the south of the classical meningitis belt [11] and to the north of South Africa, a key economic partner in terms of trade and workforce movement. It has a high HIV prevalence and in 2007, the estimate of those living with HIV infection in Malawi was approximately 900,000 [12]. In Blantyre, the prevalence of HIV infection in paediatric inpatients is approximately 19% overall [13], and 34% in children with bacterial meningitis [14]. Adult inpatient HIV prevalence is approximately 70% [15], reaching up to 90% in meningitis cases [6], [16], [17].

Free antiretroviral therapy (ART) has been provided in Malawi since 2004 through the support of the Global Fund [18]. By the end of 2009, 271,105 HIV-infected individuals had been registered on the national ART programme of which, 198,846 patients were alive and on ART at 377 ART clinics in Malawi [19]. Alongside the rapid ART scale-up, cotrimoxazole prophylaxis has been widely implemented. Although registration of cotrimoxazole prophylaxis did not begin until 2006, by 2010 there were over 250,000 patients registered [20].

It has been reported in the United States and South Africa that ART introduction has led to a reduction in the burden of IPD [21], [22]. Antibiotic resistance complicates the clinical management of invasive pneumococcal disease (IPD), particularly in resource-poor settings such as sub-Saharan Africa (SSA) where resistance to a range of commonly used antibiotics has been reported [23], [24], [25], [26], [27], [28], [29] and the dissemination of pandemic multidrug resistant (MDR) clones is a major global health concern [25], [27], [30]. Cotrimoxazole prophylactic treatment of HIV-infected individuals has the potential to amplify bacterial resistance [31], [32], [33]. We therefore analysed 10 years of surveillance data at the Queen Elizabeth Central Hospital (QECH), the only government-funded hospital serving the largest city in Malawi, Blantyre. We sought to investigate whether there has been a change in the frequency and seasonal pattern of IPD as the national ART program has scaled-up; and to determine whether cotrimoxazole prophylaxis has led to an increase in resistance to this and other frequently used antibiotics.

## Methods

### Setting

QECH is a 1250-bed government funded central hospital that provides primary to tertiary care and has an average admission rate of 50,000 patients per year (see below). QECH serves a population of approximately 1 million including the city of Blantyre, the surrounding townships, and outlying villages.

### Patients and surveillance criteria for invasive bacterial infection

During the period studied, all adult general medical admissions (15-years or older) presenting with fever (axillary temperature >37.5°C) or clinical evidence of sepsis had a recommended 5-10-mL of blood drawn for culture. Lumbar punctures (LP) were performed when there was a clinical suspicion of meningitis. Malaria is a common cause of febrile illness among children in Malawi, and all children first had a thick blood film obtained from a capillary sample examined for malaria parasites. Blood culture (1-mL) or LP were reserved for febrile children who had a negative thick film for malaria parasites and no obvious clinical focus of infection (such as pneumonia), children considered severely ill with sepsis or meningitis regardless of thick film result, and patients who failed to respond to initial treatment for malaria and remained febrile. During 2000, aerobic blood culture was performed using a manual culture system.

### *S. pneumoniae* isolation and identification

Blood was inoculated into a single 50-mL (adult) or 20-mL (paediatric) bottle of brain heart infusion broth with sodium polyanethol sulphonate (E&O laboratories, UK) and was incubated at 37°C in air for 7 days, with routine subcultures onto sheep blood agar in 5% CO_2_ at 24-h, 48-h, and 7 days. Turbid bottles were examined by Gram stain and subcultured onto appropriate media. From December 2000 onwards the same volume of blood was cultured using the BacT/Alert 3D automated system (BioMerieux, UK). All isolates were identified using standard diagnostic techniques [34]. Cerebrospinal fluid (CSF, 5-10-mL from adults and 1-2-mL from children) was analysed by Gram stains if the white blood cell (WBC) count was >10/mm^3^. All samples were cultured on sheep blood agar for 48-h under aerobic and microaerophilic (candle jar) conditions. Organisms were identified using standard methods including alpha-haemolysis and optochin sensitivity [34].

All diagnostic testing and quality control was performed in the laboratories of the Malawi-Liverpool-Wellcome Trust Clinical Research Programme (MLW) as part of a routine service provided to support QECH. The MLW laboratory participates in a number of internationally recognised quality control programmes. During 2004–2006, two large clinically based studies situated in QECH provided enhanced *S. pneumoniae* surveillance in adults and focused heavily on improved sample collection practices, particularly blood culture collection [8], [35].

### Susceptibility Testing

Antibiotic susceptibilities to ceftriaxone, chloramphenicol, cotrimoxazole, erythromycin, oxacillin, and tetracycline were determined on all isolates by disc testing (Oxoid, UK) using the British Society for Antimicrobial Chemotherapy (BSAC) sensitivity method for direct cultures [36]. At QECH isolates are reported as sensitive, resistant or of intermediate susceptibility to each antibiotic tested based on zone size measurement.

### National HIV intervention strategies

Data on ART scale-up and the cotrimoxazole prophylaxis register was obtained from the Malawi Ministry of Health [20], [37].

### Rainfall and Temperature

Although there a few detailed reports, IPD is widely believed to be seasonal in SSA. To establish whether this seasonality has been retained over the surveillance period, rainfall and temperature data were obtained from the Department of Climate Change and Meteorological Services, Malawi for the two nearest meteorological stations to Blantyre (Chileka and Chichiri). Mean rainfall and mean temperature per month for the two areas were used to obtain estimates for Blantyre.

### Ethical Approval

The isolates characterised in this study are all clinical isolates from CSF and blood culture specimens obtained from Malawian adults and children as part of routine clinical diagnosis and management when they were admitted to QECH. The main ethical issue relates to specific consent for detailed characterisation of an isolate from a clinical specimen taken from a patient on clinical grounds. This requirement was waived by the University of Malawi, College of Medicine Research & Ethics Committee because the characterisation formed part of routine clinical management. The data are therefore published with the approval of the Research & Ethics Committee and conform to institutional guidelines.

## Results

The MLW laboratory processed 99,741 (50,110 adult; 49,631 paediatric) blood cultures and 39,694 (19,213 adult; 20,481 paediatric) CSF cultures from adults and children admitted to QECH, Blantyre, Malawi with suspected severe bacterial infection between 1^st^ January 2000 and 31^st^ December 2009. During the same period, a total of 4445 pneumococcal isolates were obtained. 1837 were from children: 885 (19.9%) from blood and 952 (21.4%) from CSF. 2608 were from adults: 1813 (40.8%) from blood and 795 (17.9%) from CSF. In 80 illness episodes, pneumococci were isolated from both a blood and a CSF sample from the same individual.

The median (IQR) ages were 0.8 (0.2–3.4) and 31 (26–39) years respectively for paediatric and adult patients with pneumococcal isolates. Hospital admission data was only available from 2002 onwards. During this period, admissions remained at consistent level of around 50,000 per year. The lowest yearly admission rate was 45,896 in 2009 and the maximum was 58,414 in 2003. In the same period the number of blood cultures collected from those admitted averaged 10,000 per year. The minimum collection was 8033 in 2009 and the maximum was 12,430 in 2003.

**Figure 1:**
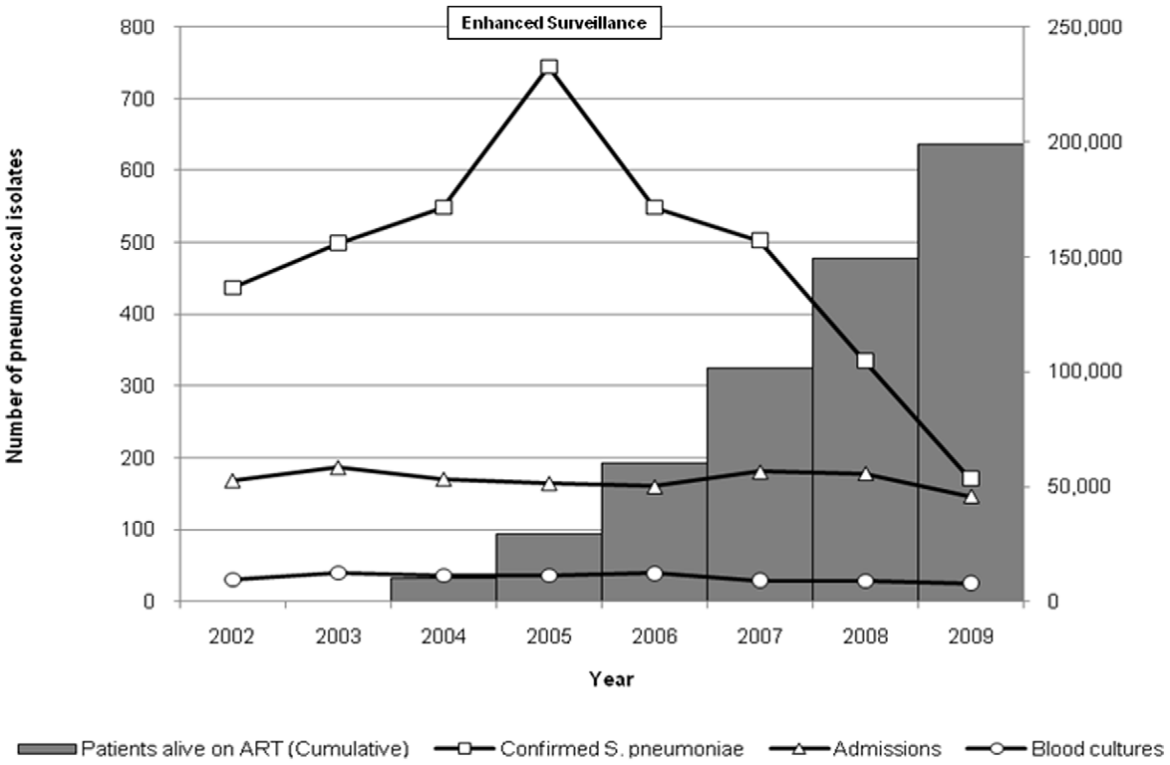
Trends in pneumococcal invasive disease and ART scale-up from 2002–2009 amongst adults and children. Data are derived from blood and CSF cultures.

There was a decline year on year of microbiologically confirmed IPD from a peak of 744 cases in 2005, during a period of enhanced surveillance, to 171 cases in 2009 which coincided with a year on year scale up of ART provision (Figure 1). There was a negative correlation between ART scale-up and the decline in microbiologically confirmed invasive pneumococcal disease (Pearson's correlation r = −0.91; p<0.001) over this time period.

**Figure 2:**
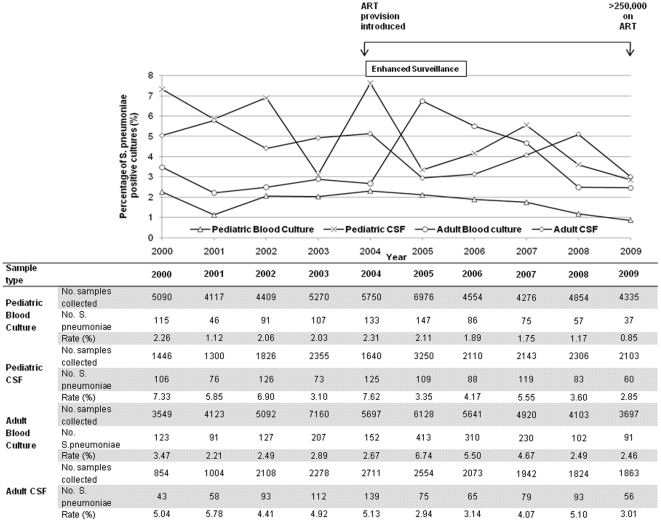
Pneumococcal recovery rates 2000–2009.

The blood culture recovery rate per blood culture taken year on year peaked in paediatric and adult samples at 2.31% in 2004 and 6.74% in 2005 respectively. By 2009 the rate had dropped to 0.85% and 2.46% respectively. The CSF recovery rate per CSF sample peaked in paediatric and adult samples at 7.62% in 2004 and 5.78% in 2001 respectively. By 2009 the rate had dropped to 2.85% and 3.01% respectively (Figure 2).

**Figure 3:**
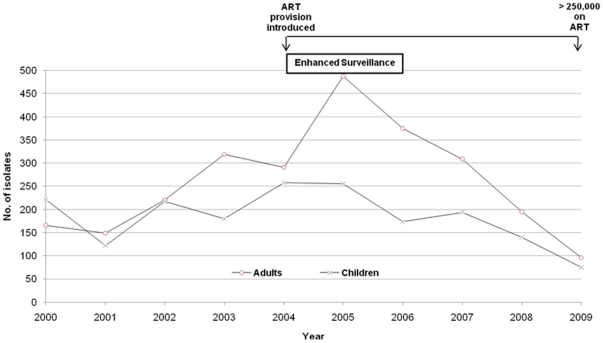
Invasive pneumococcal infections from 2000–2009 separated into adult and pediatric disease. Data are derived from blood and CSF cultures.

**Figure 4:**
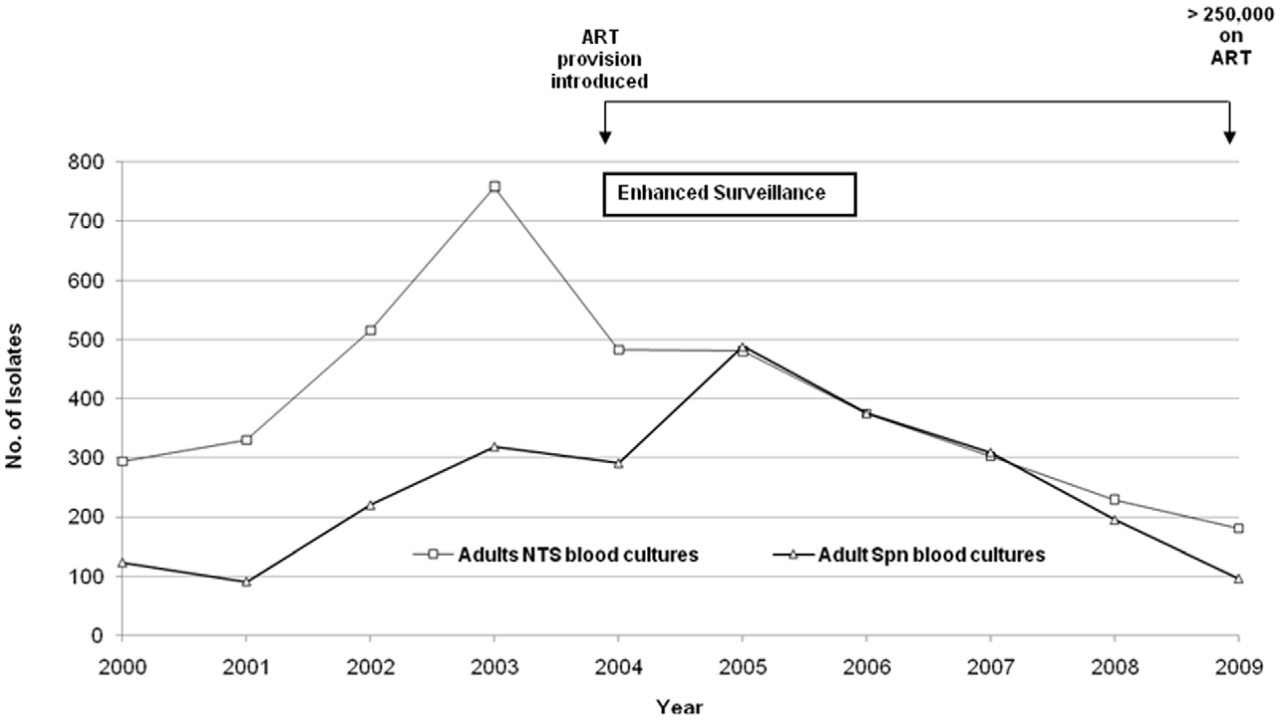
Comparison between pneumococcal and non-typhoidal salmonellae (NTS) blood cultures amongst adults, 2000–2009.

There was a decrease in the number of confirmed adult and paediatric pneumococcal infections from 2005 through 2009 (Figure 3). The decline in adults was the most marked from 488 cases in 2005 to 96 cases in 2009. A decrease year-on-year was also observed in the number of non-typhoidal salmonellae (NTS) isolates from adult blood cultures. NTS decreased from 758 cases in 2003 to 180 cases in 2009 (Figure 4).

### Seasonality

**Figure 5:**
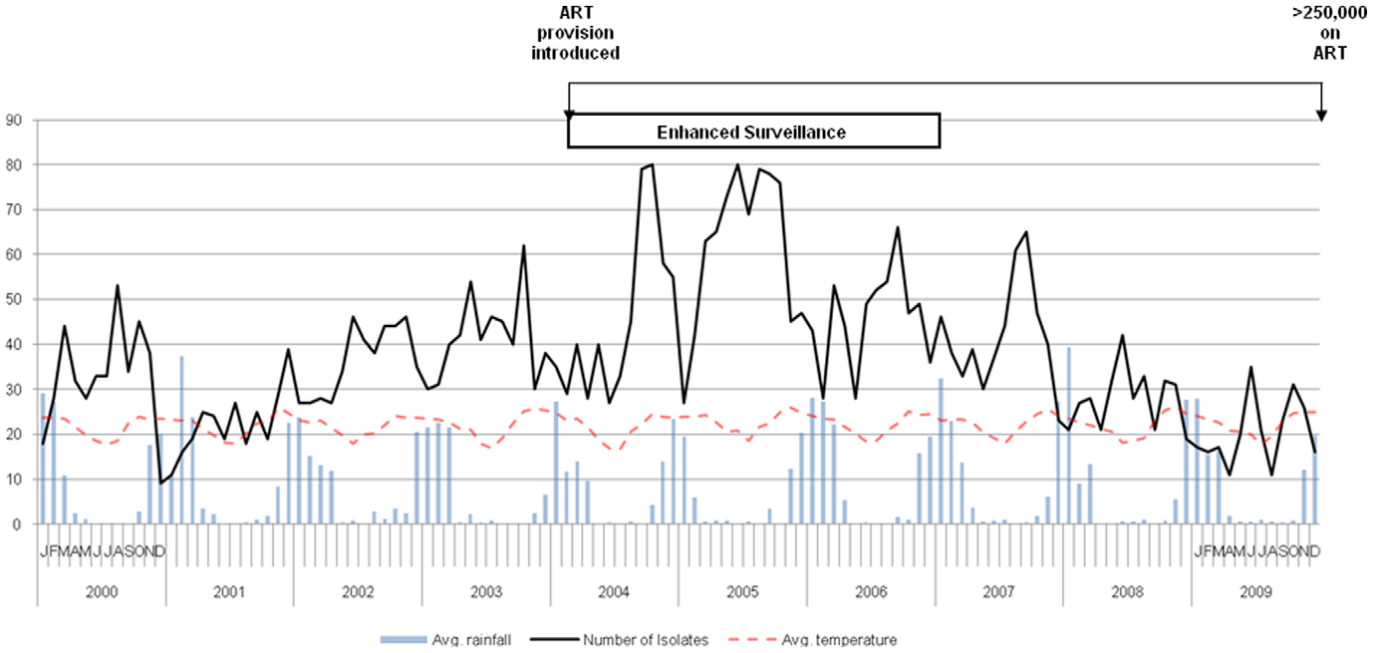
The relationship between seasonal rainfall and invasive pneumococcal disease among adults and children, by month, 2000–2009. Data are derived from blood and CSF cultures.

Mean temperatures in Blantyre over the decade ranged from 17–25°C, but the pattern of seasonal variation remained stable according to season, peaking in October/November with the coldest and driest months being June/July (Figure 5). The peak rainfall was observed in the months of January-March. The incidence of pneumococcal isolation peaked during the colder, drier months. Since 2005 this relationship has become more pronounced. Seasonality was present both in adults who had a higher HIV seroprevalence, and children, who had a relatively lower HIV seroprevalence (data not shown).

### Antibiotic resistance patterns

**Figure 6:**
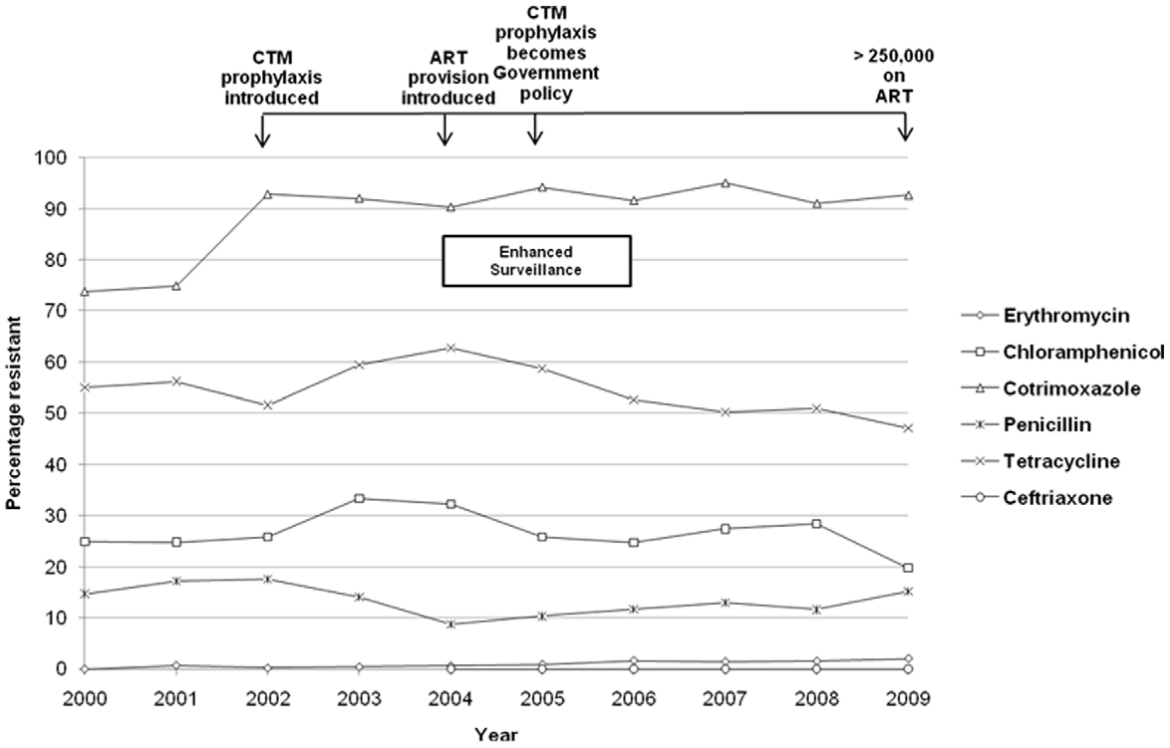
Antibiotic resistance to individual drugs from 2000–2009.

Resistance to the six first line antibiotics at QECH by year was recorded routinely throughout the study period (Figure 6). There was no reported resistance to ceftriaxone since its introduction. Chloramphenicol resistance has remained within the range of 20–33%. Cotrimoxazole resistance was already high at around 74% when data collection began, and it increased by almost 20% during 2002, since when it has remained consistently above 90%. The increase in cotrimoxazole resistance levels was not associated with increased resistance to other drugs (Table 1). Erythromycin resistance was consistently below 2%. Penicillin resistance remained within the range of 9–18% over the decade, and has been stable at approximately 10% since 2005. Tetracycline resistance varied between 50–63% throughout the decade. In Malawi, the combination of penicillin and chloramphenicol is a commonly used empirical regimen for suspected IPD in adults. Since 2000 the rate of combined resistance to both drugs remained between 1–2% of all microbiologically confirmed pneumococcal infections (data not shown), resistance to either of the drugs was between 35–45% over the decade. Multi-drug resistance (resistance to 3 or more antibiotics) has remained constant at almost one-third of isolates (range 25–37%, see Table 1).

**Table 1:** Relationship between cotrimoxazole resistance and resistance to other commonly used antibiotics in Malawi: erythromycin, chloramphenicol, penicillin, tetracycline or cetriaxone.

Resistance Combinations	Resistance rates (%)									
	2000	2001	2002	2003	2004	2005	2006	2007	2008	2009
Cotrimoxazole (CTM)	73.8	74.9	92.8	92.0	90.3	94.2	91.6	95.0	91.0	92.6
CTM + 1 other antibiotic	27.4	30.1	31.2	33.2	33.1	33.7	27.8	25.8	25.3	27.7
CTM + 2 other antibiotics	29.4	25.4	33.4	37.4	34.7	30.0	37.2	31.3	32.0	28.4
CTM + 3 other antibiotics	0.9	1.2	0.8	0.9	0.6	0.5	1.7	1.0	0.9	0.0
CTM + 4 other antibiotics	0.0	0.0	0.0	0.0	0.0	0.0	0.37	0.0	0.0	0.0

## Discussion

Pneumococcal infections place a high social, health and economic burden on the infrastructure of resource-poor countries such as Malawi [38]. The potential for the emergence of MDR *S. pneumoniae* [39] threatens to further undermine these fragile systems. Our hospital-based surveillance data shows that in the context of the rapid scale-up of ART nationally, there has been a concurrent decline in the number of invasive pneumococcal infections detected and that importantly with the exception of cotrimoxazole, the frequency of antibiotic resistant isolates, including MDR strains, has remained relatively constant. In particular, penicillin resistance amongst pneumococcal isolates was 15.1% in 2009 compared with 14.7% in 2000. This compares favourably with some other African countries where resistance has been reported in excess of 40% [31], [32]. Long term surveillance is a difficult undertaking but our data emphasise that it is vital to maintain existing and create new sentinel surveillance sites particularly in the era of vaccine introduction and with the potential for serotype replacement and the emergence of resistant strains [40].

HIV has a major impact on the frequency and mortality of pneumococcal disease in SSA [41], [42]. HIV-1 infection is linked to higher rates of pneumococcal colonisation [9], [31], [43], [44], is associated with a different pattern of serotypes and higher rates of antibiotic resistance [5], [7], [17], [45], [46]. Following the introduction of ART in Malawi in 2004, we have observed an association between ART scale-up and the decline in microbiologically confirmed invasive pneumococcal disease over the 6 year period at QECH (Pearson's correlation r = −0.91; p<0.001). These data thus add to the increasing body of evidence that widespread implementation of ART in national programmes will reduce the burden of IPD [22], [47].

Adults have a higher proportion of HIV co-infection among patients with IPD compared to children. Any observation regarding the effect of ART use would therefore be more likely in adults where the impact of HIV is greatest and the scale-up of ART has been most effective [21]. Indeed, at QECH we observed a larger decline in IPD in adults than in children (Figure 3). To further support the relationship between ART scale-up and a decrease in IPD, we also compared trends in NTS blood culture isolates in adults (Figure 4), a major HIV-associated pathogen in Malawi and elsewhere in SSA [24]. We found that from 2005 through 2009 there was a consistent decrease in recovery of NTS from adult blood cultures (Figure 3 and 4), suggesting that the provision of free ART is impacting on IPD and HIV-related bacteraemia rate in Malawi in general.

Alternate explanations for this decline in IPD admissions include changes in the socio-demographic circumstances of the catchment population over the time course of the study, such as improved access to healthcare, an effect that has been observed elsewhere [48]. Specific research studies may also have had an impact on the decline we observed. In 2004–2007, two large clinical studies situated in the adult wards of QECH provided enhanced pneumococcal surveillance through consistent efforts to improve sample collection practices [17], [35], particularly in blood culture collection. The termination of these studies may have contributed to the decline in IPD numbers by decreased ascertainment. However, a decline in the isolation to sample number ratio particularly for CSF samples supports the view that this is not simply an ascertainment effect. Environmental conditions may have also impacted on IPD rates through the progressive warming of colder months. IPD is a well-described winter disease associated with the colder months of the year in the Northern hemisphere [49], [50], [51]. In SSA, IPD is associated with the dry season, which also tends to coincide with the colder months [52]. However, during ART scale-up IPD remained seasonal, associated with the colder months and there were no significant changes in average seasonal temperatures over the decade to explain the decline in the frequency (Figure 5). This data emphasises that surveillance needs to be conducted year-round and that for planning purposes the greatest burden of disease is in the cold dry months.

The main benefit of cotrimoxazole prophylaxis in SSA is believed to be the prevention of bacterial pneumonia, diarrhoea and malaria infections [53], [54]. This effect appears to be maintained despite high prevalence of cotrimoxazole resistance in clinical isolates [55], [56], [57]. In 2005, cotrimoxazole prophylaxis for HIV-infected groups became national policy and a gradual process of scale-up to all government hospitals was started. However in QECH the availability of cotrimoxazole for prophylactic use was irregular until 2007. Cotrimoxazole resistance was already high and increased to almost universal levels from 2002 (Figure 6), prior to its wide spread use as a prophylactic agent. It is unclear if the increase in cotrimoxazole resistance was linked to policy decisions, but our data would suggest that cotrimoxazole prophylaxis scale-up has not driven resistance to the other available antibiotics which is reassuring (Table 1). Indeed our data does not support an immediate policy change regarding cotrimoxazole prophylaxis but in the context of widespread sulfadoxine/pyrimethamine malaria resistance does suggest that further evaluation of its continued efficacy is required.

This survey also provides surveillance information on antibiotic resistance trends in invasive pneumococcal isolates, which is lacking from many areas across SSA. The frequency of pneumococcal resistance to other commonly used antibiotics in Blantyre has remained stable over the decade. In Malawi, 50% of clinical pneumococcal isolates were previously reported as tetracycline non-susceptible [24] and that has remained more or less the same. Similarly, resistance rates for ceftriaxone, chloramphenicol, penicillin and erythromycin have remained largely unchanged. The level of macrolide resistance has been increasing worldwide [58], [59] yet this has not been observed in QECH, where erythromycin resistance remained very low at less than 2%. This may be a consequence of restricted availability of macrolides locally, in particular of the newer agents such as azithromycin and clarithromycin. Ceftriaxone was introduced in 2004 as a first line antibiotic and from 2004–2007 its use was tightly controlled. Since 2007 it has become more widely available for empirical treatment of sepsis and meningitis. Although current pneumococcal isolates are uniformly sensitive to ceftriaxone as determined by internationally accepted breakpoints [36], it is of concern that from 2009 we have begun to detect the first indications of an increase in ceftriaxone MIC (from 0.0016 µg/ml to 0.125 µg/ml, unpublished data). These subtle changes are the subject of further surveillance and molecular analysis. Patterns of resistance in other countries in the region may be different because of differing patterns of antibiotic prescription and drug usage [60]. In Malawi, antibiotics are only available through prescription and this policy may have contributed to limiting the increase in pneumococcal resistance rates (Figure 6). Only cotrimoxazole and tetracyclines are widely available in the informal sector.

There are several potential limitations to this study, limitations common to long term surveillance activity. Firstly the period of surveillance stretches over ten years. During this time sampling and processing guidelines have remained consistent but there has been considerable turnover of clinicians and laboratory staff. Secondly, we do not have data on HIV status, ART usage or cotrimoxazole prophylaxis for all individual IPD admissions, and therefore the impact of ART and cotrimoxazole prophylaxis can only be inferred by temporal association. Thirdly, the observations reported here are from until recently, the only long term comprehensive surveillance site in Malawi and may not be representative of the whole country. Fourthly, the microbiological database is unable to define the potential impact of recurrent admissions. We know that recurrent IPD disease in HIV-infected individuals is common. However, we know that ART and cotrimoxazole use will reduce the risk of recurrent disease [17]. We are not able to define the relative contribution of this to the lower number of IPD cases. Finally, there has been a small but steady rise in the socioeconomic status of the Malawian population during the surveillance period [61]. Such change may in itself contribute to pneumonia-related health outcomes [48]. Although problematic in large urban populations, we are currently undertaking community-based denominator surveys to further strengthen our observation and provide a robust estimate of IPD incidence in preparation for the imminent introduction of conjugate pneumococcal vaccine into the expanded programme of immunisation (EPI) in Malawi.

### Conclusion

We have shown that the scale-up of free ART coincided with a decrease in IPD. Whether ART scale up directly contributed to the decrease in IPD is not certain but our data does demonstrate a correlation between scale-up and decline in IPD. The introduction of cotrimoxazole prophylaxis in HIV-infected groups has not coincided with an increase in pneumococcal cotrimoxazole-resistance which has been almost universal since 2002. Antibiotic resistance rates in general have remained stable over the past decade. This data emphasises the well documented need for improved infectious disease surveillance across the region, but more specifically it will be vital for monitoring the effects of the introduction of pneumococcal conjugate vaccine as it is introduced into national immunisation programmes across SSA, and the monitoring of strategies to limit antimicrobial resistance to this important pathogen.

## References

[1] Embry A, Hinojosa E, Orihuela C (2007). Regions of Diversity 8, 9 and 13 contribute to Streptococcus pneumoniae virulence.. BMC Microbiology.

[2] Klein DL (1999). Pneumococcal disease and the role of conjugate vaccines.. Microb Drug Resist.

[3] Poland GA (1999). The burden of pneumococcal disease: the role of conjugate vaccines.. Vaccine.

[4] Spratt BG, Hanage WP, Brueggemann AB, E.I T (2004). Evolutionary and population biology of *Streptococcus pneumoniae*.. The pneumococcus.

[5] Gilks CF, Ojoo SA, Ojoo JC, Brindle RJ, Paul J (1996). Invasive pneumococcal disease in a cohort of predominantly HIV-1 infected female sex-workers in Nairobi, Kenya.. Lancet.

[6] Gordon S, Walsh A, Chaponda M, Gordon M, Soko D (2000). Bacterial Meningitis in Malawian Adults: Pneumococcal Disease is Common, Severe, and Seasonal.. Clinical Infectious Diseases.

[7] Gordon SB, Chaponda M, Walsh AL, Whitty CJ, Gordon MA (2002). Pneumococcal disease in HIV-infected Malawian adults: acute mortality and long-term survival.. Aids.

[8] Carrol ED, Guiver M, Nkhoma S, Mankhambo LA, Marsh J (2007). High pneumococcal DNA loads are associated with mortality in Malawian children with invasive pneumococcal disease.. Pediatr Infect Dis J.

[9] Madhi SA, Petersen K, Madhi A, Wasas A, Klugman KP (2000). Impact of human immunodeficiency virus type 1 on the disease spectrum of Streptococcus pneumoniae in South African children.. Pediatr Infect Dis J.

[10] Molyneux EM, Walsh AL, Forsyth H, Tembo M, Mwenechanya J (2002). Dexamethasone treatment in childhood bacterial meningitis in Malawi: a randomised controlled trial.. Lancet.

[11] Riou JY, Djibo S, Sangare L, Lombart JP, Fagot P (1996). A predictable comeback: the second pandemic of infections caused by Neisseria meningitidis serogroup A subgroup III in Africa, 1995.. Bull World Health Organ.

[12] UNAIDS (2008). Epidemiological Fact Sheet on HIV and AIDS:.

[13] Rogerson SR, Gladstone M, Callaghan M, Erhart L, Rogerson SJ (2004). HIV infection among paediatric in-patients in Blantyre, Malawi.. Trans R Soc Trop Med Hyg.

[14] Molyneux EM, Tembo M, Kayira K, Bwanaisa L, Mweneychanya J (2003). The effect of HIV infection on paediatric bacterial meningitis in Blantyre, Malawi.. Arch Dis Child.

[15] Lewis DK, Callaghan M, Phiri K, Chipwete J, Kublin JG (2003). Prevalence and indicators of HIV and AIDS among adults admitted to medical and surgical wards in Blantyre, Malawi.. Trans R Soc Trop Med Hyg.

[16] Scarborough M, Gordon SB, Whitty CJ, French N, Njalale Y (2007). Corticosteroids for bacterial meningitis in adults in sub-Saharan Africa.. N Engl J Med.

[17] French N, Gordon SB, Mwalukomo T, White SA, Mwafulirwa G (2010). A trial of a 7-valent pneumococcal conjugate vaccine in HIV-infected adults.. N Engl J Med.

[18] Harries AD, Zachariah R, Jahn A, Schouten EJ, Kamoto K (2009). Scaling Up Antiretroviral Therapy in Malawi-Implications for Managing Other Chronic Diseases in Resource-Limited Countries.. JAIDS Journal of Acquired Immune Deficiency Syndromes.

[19] Malawi Antiretroviral Treatment Programme (2010). Quarterly Report: Results up to 31st December 2009..

[20] Jahn A (2010). Data on ARV scale up and Cotrimoxazole prophylaxis register.

[21] Heffernan RT, Barrett NL, Gallagher KM, Hadler JL, Harrison LH (2005). Declining incidence of invasive Streptococcus pneumoniae infections among persons with AIDS in an era of highly active antiretroviral therapy, 1995-2000.. J Infect Dis.

[22] Nunes MC, von Gottberg A, de Gouveia L, Cohen C, Moore DP (2010). The impact of antiretroviral treatment on the burden of invasive pneumococcal disease in South African children: a time series analysis.. AIDS.

[23] Adegbola RA, Hill PC, Secka O, Ikumapayi UN, Lahai G (2006). Serotype and antimicrobial susceptibility patterns of isolates of Streptococcus pneumoniae causing invasive disease in The Gambia 1996-2003.. Trop Med Int Health.

[24] Gordon MA, Walsh AL, Chaponda M, Soko D, Mbvwinji M (2001). Bacteraemia and mortality among adult medical admissions in Malawi–predominance of non-typhi salmonellae and Streptococcus pneumoniae.. J Infect.

[25] McGee L, McDougal L, Zhou J, Spratt BG, Tenover FC (2001). Nomenclature of major antimicrobial-resistant clones of Streptococcus pneumoniae defined by the pneumococcal molecular epidemiology network.. J Clin Microbiol.

[26] Mudhune S, Wamae M (2009). Report on invasive disease and meningitis due to Haemophilus influenzae and Streptococcus pneumonia from the Network for Surveillance of Pneumococcal Disease in the East African Region.. Clin Infect Dis.

[27] Reinert RR (2009). The antimicrobial resistance profile of Streptococcus pneumoniae.. Clin Microbiol Infect.

[28] Wolter N, von Gottberg A, du Plessis M, de Gouveia L, Klugman KP (2008). Molecular basis and clonal nature of increasing pneumococcal macrolide resistance in South Africa, 2000-2005.. Int J Antimicrob Agents.

[29] van Oosterhout JJ, Laufer MK, Graham SM, Thumba F, Perez MA (2005). A community-based study of the incidence of trimethoprim-sulfamethoxazole-preventable infections in Malawian adults living with HIV.. J Acquir Immune Defic Syndr.

[30] Lynch JP, Zhanel GG (2009). Streptococcus pneumoniae: does antimicrobial resistance matter?. Semin Respir Crit Care Med.

[31] Gwanzura L, Pasi C, Nathoo KJ, Hakim J, Gangaidzo I (2003). Rapid emergence of resistance to penicillin and trimethoprim-sulphamethoxazole in invasive Streptococcus pneumoniae in Zimbabwe.. Int J Antimicrob Agents.

[32] Kariuki S, Muyodi J, Mirza B, Mwatu W, Daniels JJ (2003). Antimicrobial susceptibility in community-acquired bacterial pneumonia in adults.. East Afr Med J.

[33] Liebowitz LD, Slabbert M, Huisamen A (2003). National surveillance programme on susceptibility patterns of respiratory pathogens in South Africa: moxifloxacin compared with eight other antimicrobial agents.. J Clin Pathol.

[34] Barrow G, Feltham RKA, Steele Ca (1993). Manual for the identification of medical bacteria..

[35] Mtunthama N, Gordon SB, Kusimbwe T, Zijlstra EE, Molyneux ME (2008). Blood culture collection technique and pneumococcal surveillance in Malawi during the four year period 2003-2006: an observational study.. BMC Infect Dis.

[36] (2001). BSAC disc diffusion method for antimicrobial susceptibility testing.:British Society of Antimicrobial Chemotherapy (BSAC) working party.

[37] Malawi Go, Health Mo (2010). Quarterly HIV Programme Report..

[38] All-Party Parliamentary Group on Pneumococcal Disease Prevention in the Developing World (2008). Improving global health by preventing pneumococcal disease.. British Parliament.

[39] Jones RN, Jacobs MR, Sader HS. Evolving trends in Streptococcus pneumoniae resistance: implications for therapy of community-acquired bacterial pneumonia.. Int J Antimicrob Agents.

[40] Dagan R (2009). Impact of pneumococcal conjugate vaccine on infections caused by antibiotic-resistant Streptococcus pneumoniae.. Clin Microbiol Infect.

[41] Feikin DR, Jagero G, Aura B, Bigogo GM, Oundo J (2010). High rate of pneumococcal bacteremia in a prospective cohort of older children and adults in an area of high HIV prevalence in rural western Kenya.. BMC Infect Dis.

[42] Gill CJ, Mwanakasale V, Fox MP, Chilengi R, Tembo M (2008). Impact of human immunodeficiency virus infection on Streptococcus pneumoniae colonization and seroepidemiology among Zambian women.. J Infect Dis.

[43] French N, Watera C, Moi K, Whitworth J, Gilks CF (2004). Pneumococcal carriage is higher in Ugandan adults infected with HIV than in uninfected, shows seasonal variation and is directly associated with pneumococcal disease rates.. 4th International Symposium on Pneumococci and Pneumococcal Diseases.

[44] Medina MJ, Greene CM, Gertz RE, Facklam RR, Jagero G (2005). Novel antibiotic-resistant pneumococcal strains recovered from the upper respiratory tracts of HIV-infected adults and their children in Kisumu, Kenya.. Microb Drug Resist.

[45] Crewe-Brown HH, Karstaedt AS, Saunders GL, Khoosal M, Jones N (1997). Streptococcus pneumoniae blood culture isolates from patients with and without human immunodeficiency virus infection: alterations in penicillin susceptibilities and in serogroups or serotypes.. Clin Infect Dis.

[46] Gilks CF (1997). Royal Society of Tropical Medicine and Hygiene meeting at Manson House, London, 12 December 1996. HIV and pneumococcal infection in Africa. Clinical, epidemiological and preventative aspects.. Trans R Soc Trop Med Hyg.

[47] Klugman KP, Madhi SA, Feldman C (2007). HIV and pneumococcal disease.. Curr Opin Infect Dis.

[48] Dowell SF, Kupronis BA, Zell ER, Shay DK (2000). Mortality from pneumonia in children in the United States, 1939 through 1996.. N Engl J Med.

[49] Dowell SF, Whitney CG, Wright C, Rose CE, Schuchat A (2003). Seasonal patterns of invasive pneumococcal disease.. Emerg Infect Dis.

[50] Talbot TR, Poehling KA, Hartert TV, Arbogast PG, Halasa NB (2005). Seasonality of invasive pneumococcal disease: temporal relation to documented influenza and respiratory syncytial viral circulation.. Am J Med.

[51] Laurichesse H, Grimaud O, Waight P, Johnson AP, George RC (1998). Pneumococcal bacteraemia and meningitis in England and Wales, 1993 to 1995.. Commun Dis Public Health.

[52] Gessner BD, Mueller JE, Yaro S (2010). African meningitis belt pneumococcal disease epidemiology indicates a need for an effective serotype 1 containing vaccine, including for older children and adults.. BMC Infect Dis.

[53] Grimwade K, Gilks C (2001). Cotrimoxazole prophylaxis in adults infected with HIV in low-income countries.. Curr Opin Infect Dis.

[54] Grimwade K, Swingler (2003). Cotrimoxazole prophylaxis for opportunistic infections in adults with HIV.. Cochrane Database Syst Rev:.

[55] Anglaret X, Eholie S (2008). Prophylaxis with co-trimoxazole for HIV infected adults in Africa.. Bmj.

[56] Feikin DR, Feldman C, Schuchat A, Janoff EN (2004). Global strategies to prevent bacterial pneumonia in adults with HIV disease.. Lancet Infect Dis.

[57] Watera C, Todd J, Muwonge R, Whitworth J, Nakiyingi-Miiro J (2006). Feasibility and effectiveness of cotrimoxazole prophylaxis for HIV-1-infected adults attending an HIV/AIDS clinic in Uganda.. J Acquir Immune Defic Syndr.

[58] Klugman KP, Lonks JR (2005). Hidden epidemic of macrolide-resistant pneumococci.. Emerg Infect Dis.

[59] Siira L, Rantala M, Jalava J, Hakanen AJ, Huovinen P (2009). Temporal trends of antimicrobial resistance and clonality of invasive Streptococcus pneumoniae isolates in Finland, 2002 to 2006.. Antimicrob Agents Chemother.

[60] Klugman KP (2001). Antibiotic selection of multiply resistant pneumococci.. Clin Infect Dis.

[61] Government of Malawi (2008).

